# Peer Action Coordination in Middle Childhood: A Replication Null Finding on Emotion Understanding and Inhibitory Control

**DOI:** 10.3390/bs16030364

**Published:** 2026-03-04

**Authors:** Giulia Barresi, Karine Maria Porpino Viana, Tone Kristine Hermansen, Beatrice Ragaglia, Daniela Bulgarelli

**Affiliations:** 1 Department of Psychology, University of Oslo, 0373 Oslo, Norway; t.k.hermansen@psykologi.uio.no; 2 Department of Social Work, Child Welfare and Social Policy, Oslo Metropolitan University, 0130 Oslo, Norway; k.m.p.viana@psykologi.uio.no; 3 Department of Psychology, University of Turin, 10124 Turin, Italy; beatrice.ragaglia@unito.it (B.R.); daniela.bulgarelli@unito.it (D.B.)

**Keywords:** peer action coordination, emotion understanding, inhibitory control, prosocial behavior, cooperative behavior

## Abstract

Peer action coordination in middle childhood is thought to benefit from socio-cognitive abilities such as emotion understanding and inhibitory control, but empirical evidence for their role is limited. This study replicates and extends a previous study by examining whether emotion understanding and inhibitory control correlate with children’s peer action coordination in a cooperative sensorimotor problem-solving task. To test this hypothesis, 6- to 10-year-old children (*N* = 108, *M* = 8 years, 8 months, 46.3% girls, 53.7% boys) completed the Test of Emotion Comprehension and the Attention Network Task. To assess children’s performance in coordinating their actions with a peer, they were asked to complete the Labyrinth Ball Game—a sensorimotor task that they first performed individually and then together with a peer. Contrary to expectations, there was no direct association between emotion understanding or inhibitory control and children’s peer action coordination after controlling for age, gender, and individual sensorimotor skills. However, a significant interaction between age and gender revealed that older boys showed greater cooperative action coordination performance than younger boys, whereas girls’ performance remained stable across age. These findings challenge the view that individual socio-cognitive abilities straightforwardly support cooperative success, suggesting that peer action coordination in middle childhood may rely on more complex mechanisms, such as gender-specific communicative strategies or social play, rather than on emotion understanding and inhibitory control.

## 1. Introduction

Coordinating actions with others is a core social skill that becomes increasingly important in middle childhood (Saby et al., 2014). Known as peer action coordination, or joint action, it requires synchronizing one’s sensorimotor behaviors with a partner to achieve a shared goal (Sebanz et al., 2006). Unlike individual motor coordination, which integrates sensory input and motor planning within a single person, peer action coordination depends on dynamically adapting to a partner’s movements in real time, creating a shared action space greater than the sum of individual contributions (van der Wel et al., 2021). This ability is supported by socio-cognitive mechanisms such as shared representations, joint attention, and intention attribution, which allow children to anticipate and align with a partner’s behaviors (Maisto et al., 2024; Strachan & Török, 2020; Tschacher et al., 2023).

During middle childhood, from age 6 to 11, peer action coordination undergoes marked development as children refine their ability to integrate perceptual, cognitive, and social information. Around age seven, they begin to synchronize actions with partners more deliberately, showing faster and more predictable performance. By around eight years of age, they plateau individual accuracy development and adopt an online monitoring strategy—adjusting their actions dynamically to a partner’s movements rather than relying solely on pre-planned predictions (Satta et al., 2017). This developmental shift suggests that successful coordination increasingly depends on flexibility and adaptation during real-time social interaction.

One potentially relevant aspect of children’s early regulatory processes in social interactions is emotion understanding, which supports the interpretation of a partner’s emotional expressions and responses (Vesper et al., 2017). Emotion understanding is defined as the ability to comprehend the nature, causes, and consequences of one’s own and others’ emotions (Harris, 2008), and its development follows a hierarchical order. Early in life, children recognize basic emotions and external causes; by middle childhood they understand that beliefs and memories can influence emotions; and from around eight years they grasp more reflective aspects, including hidden emotions, mixed feelings, and moral implications (Pons et al., 2004). Emotion understanding has been associated with prosocial behaviors, peer acceptance, and broader social competence, suggesting it could also facilitate peer action coordination by helping children to anticipate and interpret a partner’s intentions (Caputi et al., 2012; Voltmer & von Salisch, 2017). Supporting this, Viana et al. (2020) showed that Brazilian children with higher emotion understanding, assessed through the Test of Emotion Comprehension (TEC), coordinated more effectively with peers in a cooperative sensorimotor task, beyond on the effect of age, gender, and individual sensorimotor skills. However, this finding comes from a single cultural context and has not been replicated elsewhere, leaving open the question of whether emotion understanding consistently predicts coordination in middle childhood. Moreover, the methodological design in the study of Viana et al. (2020) did not include a measure of inhibitory control, limiting the understanding of how cognitive self-regulatory processes might contribute to cooperative performance in a sensorimotor task. The present study therefore aims to replicate this previous work in a different sociocultural context and to extend it by incorporating inhibitory control as a complementary mechanism in peer action coordination.

In cooperative contexts, pausing one’s own actions to integrate feedback enhances the timing and fluidity of joint coordination (Huyder et al., 2017). Empirical work shows that inhibitory control reduces variability and improves synchrony in interactive tasks, facilitating the transition from self-centered to collaborative strategies (Meyer et al., 2015). Although inhibitory control has been linked to cooperative behaviors in preschool and early school-age children (Giannotta et al., 2011); its contribution to peer action coordination in middle childhood—and its potential interaction with emotional processes—is less explored.

Taken together, previous studies suggest that both inhibitory control and emotion understanding may support children’s ability to coordinate actions with peers. The present study examines whether emotion understanding predicts cooperative action coordination in middle childhood and whether inhibitory control contributes independently or interacts with emotion understanding in this process.

### The Present Study

The present study examines both emotional (emotion understanding) and cognitive (inhibitory control) components of peer action coordination, offering a more comprehensive view of the mechanisms underlying children’s cooperative performance. The study further examines whether inhibitory control and emotion understanding contribute independently to peer action coordination or whether inhibitory control moderates the link between emotion understanding and dyadic coordination performance.

Three hypotheses were formulated: First (H1), we expected higher emotion understanding to be associated with better peer action coordination, even after controlling for age, gender, and individual sensorimotor skills. Second (H2), we hypothesized that inhibitory control would be associated with peer action coordination, again controlling for age, gender and individual sensorimotor skills. Third (H3), we expected inhibitory control to moderate the association between emotion understanding and peer action coordination, such that dyads characterized by higher emotion understanding and higher inhibitory control would show the greatest coordination success. This expectation was grounded in the notion that inhibitory control enables flexible behavioral regulation during real-time coordination (Grazzani et al., 2018; Martins et al., 2016).

By testing these hypotheses in a sample of Italian children aged 6 to 10 years, the present study aimed to test the generalizability of the findings of Viana et al. (2020) in a different cultural context and investigate whether inhibitory control contributes uniquely or interactively to peer action coordination. The study was pre-registered on AsPredicted prior to data collection (https://aspredicted.org/mn4mr.pdf, accessed on 10 October 2023).

## 2. Materials and Methods

### 2.1. The Participants

The final sample consisted of 108 typically developing children forming 54 same-gender dyads (46.3% girls, 53.7% boys), aged 6 years 11 months to 10 years 10 months (*M* = 8 years 8 months, *SD* = 1.1), balanced across age and gender to the extent permitted by recruitment and dyad matching.

Children were recruited from four primary schools in Northern Italy, using a convenience sampling approach based on school availability. The sample included 53 children from two private schools and 65 children from two public schools. The study was conducted following ethical approval from the Norwegian Centre for Research Data (approval no.: 247851) and the local ethics committee at the Department of Psychology, University of Oslo, Norway (approval no.: 29780) where the study was coordinated. Written parental consent and child verbal assent was obtained for all participants. Most children were from middle-to-high socioeconomic backgrounds, born in Italy, and spoke Italian as their first language. The original sample was 118, however, ten children were excluded to prevent floor effect in the task and to ensure matched dyads: two failed to complete the first level of the Labyrinth Ball Game individually, and eight could not be matched for age, gender, or individual performance in the Labyrinth Ball Game.

As preregistered, the sampling plan was grounded in prior empirical work using a comparable age range and study design (Viana et al., 2020). The planned sample size was determined a priori to provide reasonable sensitivity (α = 0.05) to small-to-medium effects (*f*^2^ ≈ 0.10) for the primary main effects and interaction terms of interest; smaller effects may not be detectable with this sample.

### 2.2. Overall Procedure

The data collection consisted of three sessions conducted during school hours. In the first session, children were recorded while playing the Labyrinth Ball Game individually, to assess their individual action coordination skills. In the second session, they completed in a randomized order the Attentional Network Task (ANT; Rueda et al., 2004) to assess inhibitory control and the Italian version of the Test of Emotion Comprehension (TEC-I; Albanese & Molina, 2008) to assess emotion understanding. In this second session they also provided friendship nominations which were used to pair the dyads. To minimize competitive or cooperative biases arising from social dynamics (Sommet et al., 2015), dyads were formed by pairing children of the same gender, similar age, and comparable individual scores on the sensorimotor and emotion understanding tasks. Classroom friendship nominations were used to reduce potential advantages linked to familiarity (i.e., best friends’ prior experience coordinating during play) and to mitigate the risk of social conflict (Kuhnert et al., 2017), as close friends may display higher baseline coordination and synchronization that could bias task performance. In the third session, children were filmed while playing the Labyrinth Ball Game together with a peer. Each session lasted approximately 10–15 min.

### 2.3. Measures

#### 2.3.1. Labyrinth Ball Game (Viana et al., 2020)

**Figure 1:**
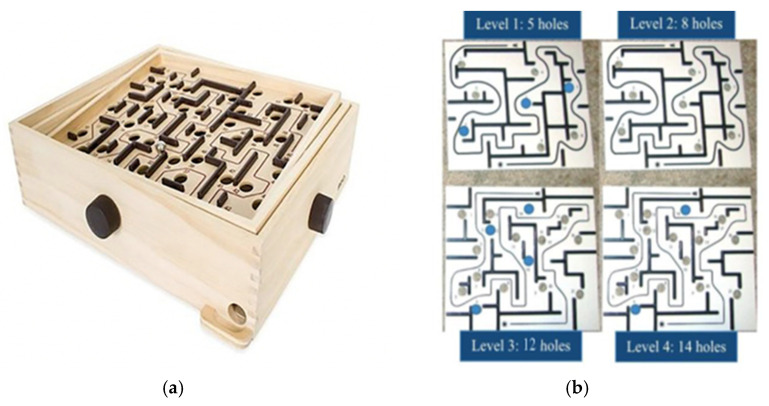
(**a**) Labyrinth ball game (BRIO-34000); (**b**) the four levels layout: level 1, 3 and 4 with some occluded holes.

Children were first tested individually and then in a cooperative condition using the Labyrinth Ball Game (Viana et al., 2020). In this task, children are challenged to maneuver a steel ball through a maze without letting it fall into holes by tilting the board with two adjustable knobs (see Figure 1). The task requires fine motor coordination and problem-solving skills.

In the individual condition, children used both hands—one on each knob—to maneuver the ball through the maze. In the cooperative condition, each child controlled one knob, requiring real-time integration of their actions and perspectives with their partner. The game consisted of four levels of increasing difficulty. The first and second levels shared the same path but included five and eight holes, respectively, whereas the third and fourth levels featured more complex layouts with 12 and 14 holes, for a total of 39 possible holes. Children were allowed up to five attempts per level; if they failed to complete the first level within five attempts, the task was discontinued to avoid frustration.

Performance was quantified based on how far the ball progressed within each level. For each attempt, the number of successfully passed holes was recorded, with higher values indicating greater progress. When a level was not completed, the score corresponded to the furthest hole reached. Rather than selecting the best attempt, performance for each level was calculated as the average across all attempts (up to five), thereby capturing consistency across trials and reducing the influence of occasional successes or failures. The final performance score was computed by summing the average scores across the four levels and dividing by the maximum possible score (39), yielding a normalized index between 0 and 1. All performances were coded from video recordings by six trained coders using standardized scoring guidelines, who practiced on pilot data (*N* = 6; not included in the final sample) to ensure consistent application of the criteria. Because the experimental data were not cross-coded, formal inter-rater reliability statistics were not calculated. Any coding ambiguities were resolved through discussion, after which each coder coded their assigned subset of data.

#### 2.3.2. Test of Emotion Comprehension (TEC; Pons & Harris, 2000)

**Figure 2:**
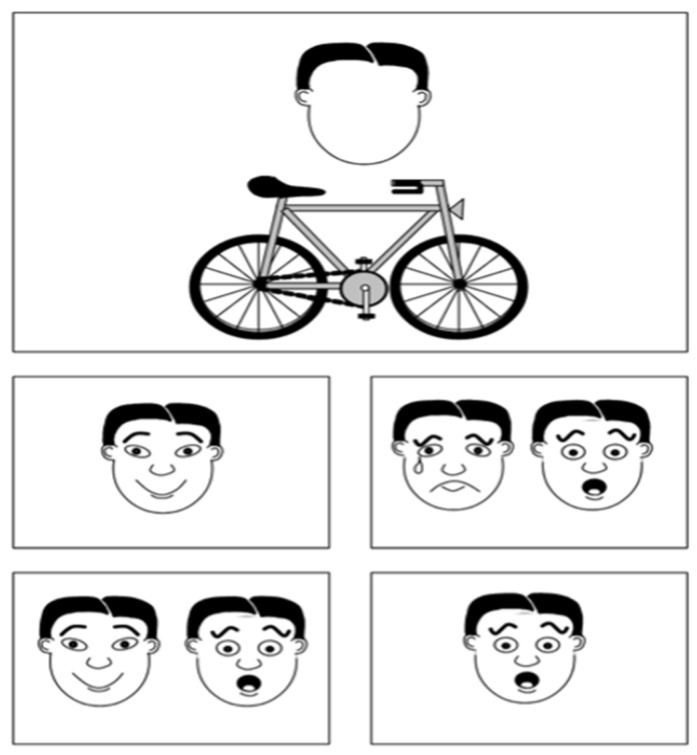
Component VIII: Mixed emotions; Table 20, of TEC-I male Italian version by Albanese and Molina (2008).

Children’s emotion understanding was assessed using the Italian version of the Test of Emotion Comprehension, standardized by Albanese and Molina (2008). The TEC evaluates both emotion recognition and knowledge (Castro et al., 2016) across nine components: recognition of basic emotions; understanding of situational, desire-, belief-, and memory-based emotions; control and concealment of emotions; mixed emotions, and moral emotions. The test consists of 23 illustrated scenarios in a picture book. For basic recognition, children identify the correct emotion among four facial expressions. For the remaining stories, the protagonist’s face is left blank, and after listening to the story, the child point at the most appropriate emotional outcome, as shown in Figure 2.

Responses were scored binarily (1 = correct, 0 = incorrect), yielding a total score ranging from 0 to 9 components mastered. Following the Italian standardization (Albanese & Molina, 2008), children’s scores can also be classified into three developmental levels of emotion understanding: the External stage (scores 1–3), reflecting understanding of observable expressions and situational causes of emotions; the Mental stage (scores 4–5-7), indicating the ability to link emotions to internal states such as beliefs and desires; and the Reflective stage (scores 6-8–9), which captures the comprehension of more complex phenomena such as mixed, moral, or self-conscious emotions. The TEC has been translated into 27 languages and demonstrates strong reliability and construct validity (Harris & Cheng, 2022).

#### 2.3.3. Attentional Network Task (ANT; Rueda et al., 2004)

**Figure 3:**
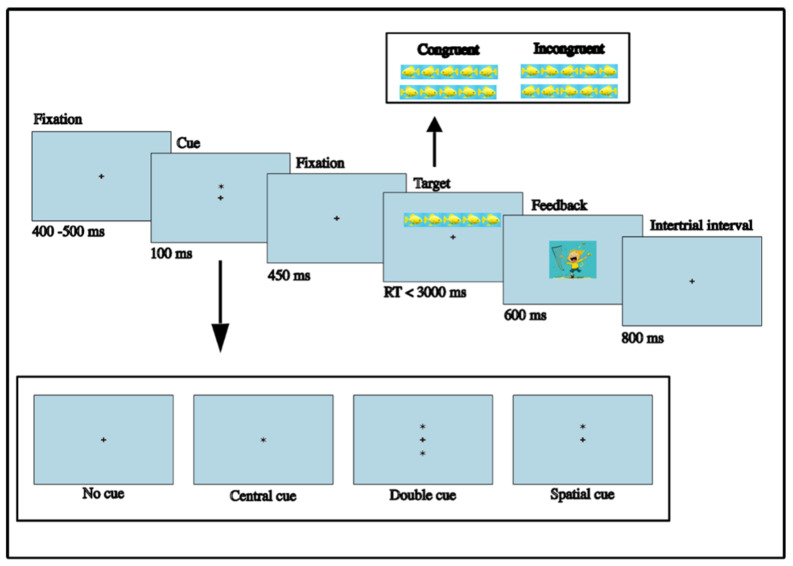
Overview of an ANT experimental trial. + is the fixation point, while * is the cue.

Inhibitory control was assessed using a child-adapted version of the Attentional Network Task, following the shortened procedure by Miljeteig (2016) and focusing only on measures of inhibitory control—defined as the ability to resolve conflicts between congruent and incongruent stimuli. Children were tested individually in a quiet room on a 15.6-inch laptop using Psytoolkit (Kim et al., 2019; Stoet, 2017). They were introduced to the task as a video game in which they helped a man catch animals. On each trial, five animals (fish or birds) appeared in a row; the child had to identify the direction of the central target animal by pressing the corresponding arrow key, ignoring the flanker animals that may or may not move in the same direction as the target. That is, on congruent trials, all animals faced the same direction, while on incongruent trials, the flanker animals faced the opposite direction of the target animals, requiring greater inhibitory control in order to identify the target direction to catch the animal. Each trial followed a fixed time sequence involving first a fixation cross (lasting 400–500 ms), followed by a brief cue as to the location of the stimuli on the screen (none, central, double, high, or low; lasting 100 ms), before finally the target array was presented (remaining on screen until a response was given or 3000 ms had elapsed). Correct and incorrect responses were followed by a brief positive or negative visual and auditory feedback, respectively. The task comprised two short practice blocks consisting of 12 trials each, followed by four experimental blocks of 32 trials each. The trials within each experimental block were presented in a randomized order, balanced across congruency and cue conditions (for details, see Figure 3).

Accuracy and reaction times (RTs) were recorded for each condition, although only RTs were used in the present analyses. No participants were excluded based on overall accuracy. Reaction times (RTs) were analyzed only for correct trials; responses faster than 150 ms or slower than 3000 ms were excluded as they likely reflected anticipatory responses or attentional lapses (Hermansen et al., 2017). RTs deviating more than ±3 *SD* from a participant’s mean were also removed to reduce the influence of non-systematic noise. Inhibitory control was operationalized as the difference between incongruent and congruent RTs, with smaller difference scores indicating better inhibitory control. Negative difference values—reflecting faster responses on incongruent than congruent trials—were retained and interpreted as reflecting individual differences in systematic anticipatory attentional strategies.

## 3. Results

### 3.1. Descriptives

Dyad-level emotion understanding scores averaged 7.50 (*SD* = 1.15, range = 4–9), out of a maximum of 9 points, indicating relatively high levels of competence in recognizing and reasoning about emotions. Although dyads were matched on emotion understanding, substantial variability remained at the dyad level (dyad-mean TEC *SD* = 1.15). Dyad-mean inhibitory control, indexed by the reaction time (RT) difference between incongruent and congruent trials in the ANT, showed a mean RT cost of 129.65 ms (*SD* = 70.67 range = −8.00–329.25 ms). Performance on the ANT indicated high task compliance, with accuracy on incongruent trials averaging 96.4% (*SD* = 6.3%). The accuracy cost (incongruent−congruent) was small (*M* = −2.55%, *SD* = 4.09), and there was no evidence of a speed–accuracy trade-off, as the association between RT cost and accuracy on incongruent trials was small and non-significant (*r* = 0.12, *p* = 0.18). For the coordination tasks, children showed an average accuracy of 57% *(SD* = 11%, range = 38–85%) in individual action coordination and 60% (*SD =* 14%, range = 30–88%) in cooperative action coordination. Only participants who successfully completed the first level were included in the analyses. In the individual condition, 76.8% of children reached the second level, 44.6% reached the third, and 14.3% completed the fourth. In the cooperative condition, 96.4% of dyads reached the second level, 73.2% reached the third, and 35.7% completed the final level. These distributions indicate that while ceiling effects were present at the easiest levels, performance variability increased substantially at higher levels.

### 3.2. Preliminary Analyses

Table 1 shows that age correlated positively with emotion understanding (*r* = 0.52, *p* < 0.001) and moderately with individual action coordination (*r* = 0.30, *p* = 0.025). That is, with increasing age, children showed higher emotion understanding and individual action coordination, as well as improved inhibitory control, reflected by smaller differences in reaction times between incongruent and congruent trials on the ANT (*r* = −0.45, *p* < 0.001). Individual action coordination was not significantly associated with emotion understanding (*r* = 0.23, *p* = 0.083) but was negatively associated with inhibitory control (*r* = −0.34, *p* < 0.001). Emotion understanding also correlated negatively with inhibitory control (*r* = −0.27, *p* = 0.045). Cooperative action coordination was not significantly correlated with individual action coordination (*r* = 0.16, *p* = 0.249), age (*r* = 0.20, *p* = 0.143), emotion understanding (*r* = 0.14, *p* = 0.315), or inhibitory control (*r* = −0.06, *p* = 0.647) (Table 1).

Gender analyses revealed that boys’ dyads outperformed girls’ dyads in both individual action coordination (boys: *M* = 0.60, *SD* = 0.11; girls: *M* = 0.52, *SD* = 0.09; *t*(54) = −2.88, *p* = 0.006) and cooperative action coordination (boys: *M* = 0.66, *SD* = 0.12; girls: *M* = 0.53, *SD* = 0.13; *t*(50) = −4.02, *p* < 0.001). No dyad gender effects emerged for emotion understanding (*t*(53) = 1.19, *p* = 0.240) or inhibitory control (*t*(47) = −0.42, *p* = 0.676).

### 3.3. Model Comparisons for Predictors of Cooperative Action Coordination

To test whether we could replicate the findings of Viana et al. (2020), we conducted a series of regression analyses focusing on the same set of predictors as in the original study. Because cooperative action coordination reflects the joint performance of both children, all analyses were conducted at the dyad level. Accordingly, predictor variables were aggregated within dyads (i.e., mean age, mean individual action coordination, mean emotion understanding, and mean inhibitory control). Gender was modeled at the dyad level, as all dyads were composed of same-gender peers.

As a baseline model (M0), we tested whether the control variables age, gender, and individual action coordination predicted peer action coordination. To assess H1 and examine whether higher emotion understanding was associated with better peer action coordination, we estimated a first model (M1) by adding emotion understanding to the baseline model and compared models M0 and M1.

Focusing on the main aim of the paper, we next tested H2 by estimating a second model (M2) in which inhibitory control was added to the baseline model and compared models M0 and M2. Finally, to test H3 and examine whether inhibitory control moderated the association between emotion understanding and peer action coordination, we estimated a third model (M3) including both emotion understanding and inhibitory control as well as their interaction term and compared models M1 and M2. As an exploratory analysis, we then estimated a fourth model (M4) including an Age × Gender interaction, which was compared against the baseline additive model (M0) as well as all previously specified models.

See Table 2 for an overview of all planned models.

The baseline model (M0: Coop ~ Age + Individual + Gender) accounted for 28.2% of the adjusted variance (*F*(3,52) = 8.21, *p* < 0.001), with age (*β* = 0.04, *p* = 0.012) and gender (*β* = 0.16, *p* < 0.001) as significant predictors, and individual action coordination being a non-significant predictor (*β* = −0.19, *p* = 0.270), contrary to our predictions. Adding emotion understanding (M1) did not significantly improve the model fit (*ΔR*^2^ = 0.00), contrary to H1. Likewise, adding inhibitory control (M2) yielded no additional explained variance (*ΔR*^2^ = −0.01), contrary to H2, and the interaction between emotion understanding and inhibitory control (M3) also failed to explain variance (*ΔR*^2^ = −0.02), contrary to H3. In contrast, including an age × gender interaction (Model 4) significantly improved model fit (*ΔR*^2^ = 0.10), yielding the lowest AIC (−83.2).

**Figure 4:**
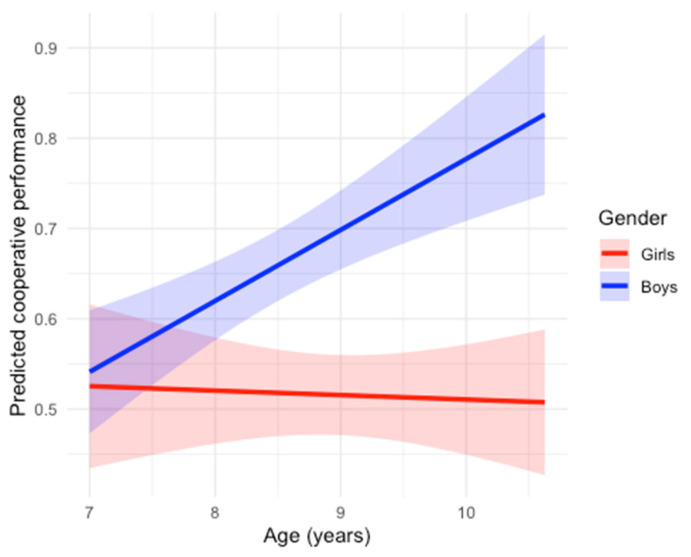
Model-predicted cooperative performance (0–1), as a function of age (in years) and dyads’ gender. Lines represent estimated marginal effects derived from the regression model, and shaded bands indicate 95% confidence intervals around the model-predicted values. Results indicate that cooperative performance increases with age in male dyads but remains stable across age in female dyads.

Importantly, because dyads were composed of same-gender peers, this interaction reflects differences between male and female dyads, rather than individual-level gender effects. The interaction was significant (*β* = 0.084, *SE* = 0.027, 95% *CI* [0.029, 0.138], *p* = 0.003). Age was not significantly associated with cooperative performance in girls’ dyads (*β* = −0.005, *p* = 0.81), whereas age was positively associated with cooperative performance in boys’ dyads (see Figure 4). Overall, the final model explained 38.4% of the adjusted variance in cooperative performance.

## 4. Discussion

This study investigated the unique and interactive contributions of children’s emotion understanding and inhibitory control to cooperative action coordination with peers. We hypothesized that both skills would be positively related to children’s cooperative performance and that inhibitory control would moderate the association between emotion understanding and coordination, such that children high in both would show the strongest outcomes in coordinating their actions with peers.

Contrary to our expectations, these hypotheses were not confirmed. That is, once age, gender, and individual performance were controlled for, neither emotion understanding nor inhibitory control predicted cooperative action coordination, and their interaction was also non-significant. Instead, the strongest predictors were age and its interaction with gender: boys’ coordination improved with age, whereas girls’ performance remained relatively stable across the sampled age range. This contrasts with the results from Viana et al. (2020), who reported emotion understanding as a reliable predictor of peer action coordination in younger children. Similarly, several studies have emphasized emotion understanding as a critical facilitator of social coordination, enabling children to anticipate partners’ intentions and synchronize their actions effectively (Grueneisen et al., 2015; Roazzi et al., 2015; Trentacosta & Fine, 2010). The absence of such an effect in our study challenges the assumption that conceptual emotion knowledge automatically enhances real-time interaction (Ramani & Brownell, 2014). Yet, our findings align with theoretical accounts suggesting a dissociation between conceptual and applied socio-emotional skills (Lucena, 2018; Meins et al., 2006; Viana et al., 2022). Even when children hold sophisticated emotional representations, the dynamic demands of joint coordination—requiring fast adaptation, shared intentionality, and complementary role assignment—may hinder the straightforward deployment of emotion understanding in action (Apperly & Butterfill, 2009; Samson et al., 2010). This interpretation is consistent with Fitzpatrick et al. (2018), who showed that emotion understanding and theory-of-mind measures predicted spontaneous but not task-driven synchrony, indicating that structured motor cooperation may depend on broader communicative and executive strategies beyond emotion comprehension alone.

Inhibitory control—although central to early cooperative success (Ciairano et al., 2007; Giannotta et al., 2011; Huyder et al., 2017)—did not predict joint action in this school-age cohort. This aligns with developmental evidence that the importance of executive inhibition for cooperation diminishes as children’s communicative and strategic competencies mature, allowing them to rely on more flexible, language-mediated approaches. Milward et al. (2017), also found that inhibitory control did not moderate the effect of theory of mind on joint actions in typically developing populations, reinforcing the idea that by middle childhood, the coordination outcomes in school-age children are not moderated by executive functioning but may instead reflect the influence of accumulated play experience, social conventions, and role negotiation strategies.

Instead, age and gender, and their interaction, significantly shaped cooperative outcomes. Older children displayed better joint coordination, which is consistent with well-established motor and social maturation trajectories that improve the speed, consistency, and predictability of interaction (Li et al., 2022; Paulus, 2016; Satta et al., 2017). The observed gender differences in peer action coordination likely reflect a convergence of sociocultural, developmental, and sensorimotor factors rather than a single underlying mechanism. From a sociocultural perspective, boys’ greater engagement in physical, goal-directed play—documented across cultures—may provide more frequent opportunities to practice fine-grained motor control, spatial coordination, and rapid adjustment to a partner’s actions, all of which are central to successful performance in the Labyrinth Ball Game (Barbu et al., 2011; Pellegrini & Smith, 2003). Developmentally, this experience-based account is consistent with the observed age × gender interaction: boys’ coordination improved steadily with age, whereas girls’ performance remained relatively stable, suggesting differences in developmental timing or accumulated play experience rather than intrinsic ability differences (Crozier et al., 2019; Hauge et al., 2023). At the sensorimotor level, prior work has reported gender differences in joint action tasks involving visuomotor integration, timing, and force modulation, which may differentially support performance in physically demanding coordination contexts such as the present task (Kaczkurkin et al., 2019; Kuhnert et al., 2017) Importantly, some children reported prior familiarity with the Labyrinth Ball Game, raising the possibility that uneven exposure—potentially shaped by gendered toy availability and play norms—may have further amplified performance differences. Together, these factors suggest that boys’ advantage reflects experience-dependent and culturally scaffolded developmental pathways rather than biologically fixed differences in cooperative capacity.

In conclusion, our findings support a developmental shift in the mechanisms underpinning peer cooperation: while younger children may rely heavily on basic executive and emotional skills, older children increasingly leverage accumulated motor experience, strategic communication, and culturally shaped play practices. This shift may explain why, in our older sample, age and gender effects emerged as the primary predictors of cooperative success, whereas emotion understanding and inhibitory control no longer exerted a strong influence.

### Limitations and Future Directions

Some methodological aspects may help explain why our findings diverged from earlier work. First, our slightly older sample compared to Viana et al. (2020)—with fewer first graders and more fifth graders—likely captured a developmental window in which emotion understanding and inhibitory control are already well consolidated (Cavioni et al., 2020; Harris & Cheng, 2022). Once children reach this stage, they rely more on refined motor and social strategies, which we did not directly assess through language or boarder social measures. Including such variables in future studies could clarify how these skills, together with emotion understanding and inhibitory control, jointly support peer action coordination. This may reduce the variability in emotion understanding and limit emotion understanding’s explanatory power for cooperative performance. This does not undermine its importance at earlier ages but suggests its influence may plateau as other strategies emerge. Secondly, because dyads were matched on emotion understanding, variability on this measure was reduced, which may have weakened its association with cooperative performance; however, matching was approximate rather than exact, and meaningful variability remained across dyads (dyad-mean TEC *SD* = 1.15, range = 4–9), so the null effect should be interpreted cautiously. Thirdly, although we matched dyads for age and individual motor skills, we did not control for within-dyad differences in inhibitory control. Prior work suggests that uneven self-regulatory abilities within a dyad can shape joint outcomes, with stronger partners sometimes compensating—or being hindered by—weaker ones (Giannotta et al., 2011). Fourthly, the highly structured experimental setting, while necessary for standardization, may have constrained the spontaneous communicative and relational strategies children typically use during free play, thereby reducing the sensitivity of emotion understanding and inhibitory control measures to individual differences relevant for cooperative performance. Our simple categorization of peers as “friends” or “neutral” likely missed the nuances of classroom social networks that influence cooperation (Ramani & Brownell, 2014). Finally, the cultural context of the sample should be considered. All participants were recruited from primary schools in Northern Italy, where children’s play experiences and gender socialization practices may be culturally shaped. As such, caution is warranted in generalizing the observed gender differences in cooperative coordination to contexts with different cultural norms; cross-cultural studies may help determine whether these patterns reflect universal developmental processes or culturally specific experiences.

Future research could address these points by including younger cohorts, when emotion understanding and inhibitory control still show significant developmental variability and may better predict cooperation. Designing tasks with mixed-gender dyads could clarify whether children adopt different coordination strategies when collaborating with same- versus opposite-gender peers. Although such designs introduce methodological challenges (e.g., accounting for familiarity and gender norms), they may reveal adaptive interaction strategies and provide a more ecologically valid picture of peer cooperation. Moreover, combining structured tasks with naturalistic observations—such as free play or classroom activities—would enhance ecological validity and reveal how children’s verbal and nonverbal communication mediate the link between conceptual socio-emotional skills and cooperative success. Qualitative studies analyzing how the interaction unfolds during cooperative coordination may also be relevant to understand how social skills might relate to the final cooperative action coordination performance.

## Figures and Tables

**Table 1:** Correlation matrix.

Variable	1	2	3	4	5
Cooperative action coordination	1.00	0.16	0.20	0.14	−0.06
Individual action coordination	0.16	1.00	0.30 *	0.23	−0.34 **
Age	0.20	0.30 *	1.00	0.52 **	−0.45 **
Emotion understanding	0.14	0.23 *	0.52 **	1.00	−0.27 *
Inhibitory control	−0.06	−0.34 **	−0.45 **	−0.27 *	1.00

Note: Values are Pearson correlation coefficients (*r*). * *p* < 0.05, ** *p* < 0.01.

**Table 2:** Model comparison for predictors of peer action coordination in the Labyrinth Ball Game.

Model	Factors	AIC	*Δ*AIC	Adjusted R^2^	*Δ*R^2^
M0	Age + Indi + Gender	−75.6	7.6	0.28	−
M1	Age + Indi + Gender + EU	−74.6	8.6	0.28	0.00
M2	Age + Indi + Gender + Inhib	−73.6	9.6	0.27	−0.01
M3	Age + Indi + Gender + Inhib * EU	−71.0	12.2	0.26	−0.02
**M4**	Age + Indi + Gender + Age * Gender	**−83.2**	**0.0**	**0.38**	**0.10**

* Age = age in months; Indi = individual sensorimotor performance at Labyrinth Ball game; Eu = TEC score as a measure of emotion understanding; Inhib = RT difference between congruent/incongruent ANT trial as a measure of inhibitory control. *Δ*AIC = difference from the lowest AIC; *Δ*R^2^ = increase in explained variance from the baseline model. The lowest AIC (M4), highlighted in bold, indicates the best fitting model.

## Data Availability

Material instructions, data and analysis script are available at https://doi.org/10.17605/OSF.IO/UB6DS.
